# The edutainment program on knowledge, perception, and uptake of cervical cancer screening among Muslim women in Southern Thailand: a quasi experimental study

**DOI:** 10.1186/s12889-024-19287-y

**Published:** 2024-07-06

**Authors:** Tassanapan Weschasat, Nuttawut Wetchasat, Montakarn Chuemchit

**Affiliations:** 1 College of Public Health Sciences Chulalongkorn University, Bangkok, 10330 Thailand; 2 Excellent Center for Health and Social Sciences and Addition Research, College of Public Health Sciences, Chulalongkorn University, Bangkok, 10330 Thailand; 3 Surat Thani Hospital, Surat Thani, 84000 Thailand

**Keywords:** Edutainment Program, Cervical cancer screening, Muslim women, Thailand

## Abstract

**Introduction:**

Cervical cancer is a significant global health concern and is the third most common cancer in women. Owing to their religious beliefs, Muslim women in Thailand are less likely to be screened for cervical cancer.

**Objective:**

This study aimed to explore how a Health Belief Model (HBM) (HBM = Health Belief Model)-Based Edutainment Program affects the knowledge, perception, and uptake of cervical cancer screening among Muslim women in Thailand.

**Methods:**

A quasi-experimental study was conducted in two rural districts of Southern Thailand with 83 Muslim women (intervention = 42, control = 41). The assessment was conducted through face-to-face interviews at baseline, post-intervention, and at 3-month follow-up. The intervention included four sessions involving video clips, folk songs, and short films. Data analysis was performed using repeated-measures ANOVA (ANOVA = Analysis of Variance) at a significance level of 0.05.

**Results:**

There were significant differences in the mean score of knowledge and perception between the intervention and control groups post-intervention and at 3-month follow-up (*p* < 0.001). The mean scores of knowledge and perception in the intervention group significantly increased post-intervention and at 3-month follow-up (*p* < 0.001). The uptake of cervical cancer screening tests in the intervention group was approximately twice as high as that in the control group (90.47% vs. 51.21%).

**Conclusion:**

The findings revealed that the Edutainment Program could improve the knowledge, perception, and uptake of cervical cancer screening among Muslim women in Thailand. In future studies, the intervention suggests testing different population groups to improve access to primary care for everyone.

**Supplementary Information:**

The online version contains supplementary material available at 10.1186/s12889-024-19287-y.

## Introduction

Cervical cancer is a significant public health issue that endangers women’s health and well-being worldwide [1]. Globally, 2,784 million women over 15 years of age are at a risk of developing cervical cancer. Annually, 569,847 women are diagnosed with the disease and 311,365 women die from cervical cancer [2]. Eighty-five percent of cervical cancer cases occur in developing countries and account for 13% of all cancer cases in females. With 20 deaths per 100,000 people, Asia has the highest mortality rate from cervical cancer [3]. There were 315,232 new cases of cervical cancer in Asia, of which 64% were in women aged 40–64 years. In this age group, 97,610 patients have died [4]. Mongolia, Indonesia, Maldives, Nepal, and Brunei are the five countries with the highest incidence rates ranging from 20.6 to 23.5 per 100,000 individuals. The incidence rates of cervical cancer in these five countries have some cases that are partly caused by the Human Papilloma Virus (HPV^1^). Southeast Asia ranks sixth among all subregions worldwide in terms of cervical cancer mortality. Thailand ranks eighth in Asia, with an incidence rate of cervical cancer cases attributable to HPV of 16.2 per 100,000 people [5]. Fortunately, cervical cancer can be prevented and treated, and early detection has reduced morbidity and mortality rates globally [6]. Papanicolaou (Pap) smear is an efficient, affordable, and effective method for examining the uterine cervix for cytological abnormalities. The incidence and mortality of invasive cancer can be reduced through screening programs [7, 8].

In Thailand, the Ministry of Public Health has been promoting the Pap smear test among women aged 30–60 years, with a regular follow-up screening interval of five years [9]. The Department of Medical Services set a target of 80% for screening and early detection among women aged 30–60 years in Surat Thani, a province in Southern Thailand. However, from 2015 to 2019, the coverage of the Pap smear test was only 47.89%. [10]. In five districts of Mueang Surat Thani, Samui, Chaiya, Tha Chana, and Ban Na Doem, the coverage of the Pap smear test was < 35%. Considering the population's religion, only 17% Muslim women had undergone the screening [10, 11].

According to a literature review, cultural beliefs, religion, reproductive history, risk behaviors, attitudes, and sociocultural norms affect Muslim women's access to services and information related to cervical screening. In addition, Muslim women experience shame during screenings, fear of losing their traditional roles as women, fear pain and infection, lack knowledge, and find screenings expensive and inaccessible [6–8, 12]. Therefore, Muslim women are at risk of missing cervical cancer screenings [8, 11]. The absence of screening results in late-stage diagnosis increases treatment burden and mortality [13–15]. Since Islam is a popular religion in Southern Thailand, it suggests that religion may be a factor in these screening discrepancies [16]. Faith, beliefs, and cultural influences can impact health behaviors, including involvement in screening initiatives. Different communities may hold specific cultural beliefs that influence their approach to screening practices. The Islamic faith may influence health behaviors related to cervical cancer screening due to beliefs in the importance of modesty and self-respect for women. These beliefs could lead them to refrain from or be hesitant to participate in screenings, which may be perceived as intrusive due to their emphasis on privacy and personal dignity [12, 17, 18]. The disparities in cervical cancer screening among Muslim women may be due to their modesty [18–20].

There are many different factors that influence cervical cancer screening, and existing interventions have not fully addressed the barriers experienced by Muslim women during screening, which contributes to the ongoing disparities in cervical cancer screening among Thai Muslim women, particularly in the Surat Thani Province. Therefore, this study aimed to investigate how edutainment affects the uptake of cervical cancer screening among Thai Muslim women in the Surat Thani Province. The intervention program was developed using t-Based Edutainment Program with video clips, folk songs, and short films. These findings can increase the knowledge, perception, and uptake of cervical cancer screening among Thai Muslim women and encourage healthcare providers to apply the edutainment approach when promoting cervical cancer screening tests to the target population.

## Materials and methods

### Study design and participants

This study applied a quasi-experimental design and was conducted in the rural areas of Southern Thailand between May and November 2021. This study was approved by the Ethics Review Committee for Research Involving Human Research Subjects, Health Science Group, Chulalongkorn University (158.3/63). Written informed consent was obtained from all participants involved in the study.

The inclusion criteria were Muslim women who had lived in Surat Thani province for more than a year, aged between 30–60 years, had never undergone a cervical cancer screening test, had been married or cohabiting, had access to healthcare services, could undergo a cervical cancer screening test, could communicate in the Thai language using the local dialect, and were willing to participate in the study. Muslim women who underwent hysterectomy with cervix removal and pregnant women were excluded.

### Sampling technique and sample size

**Fig. 1:**
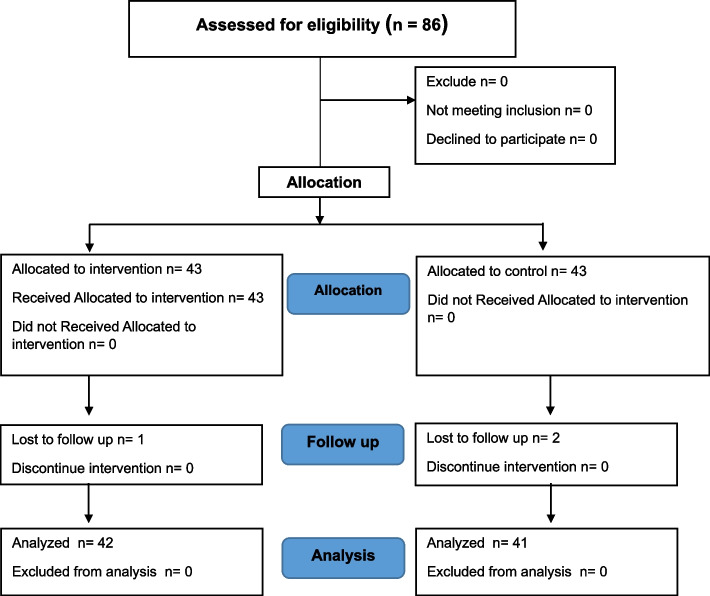
Consort diagram of the participants

The participants (Fig. 1) were selected using a two-stage random selection technique. In the first step, two of 19 districts in Surat Thani Province with the lowest Pap smear test coverage were purposively selected. Using simple random sampling, Muslim women from Mueang Surat Thani district were assigned to the intervention group, while Muslim women from Chaiya district were assigned to the control group. In the second step, the researcher checked household records accessible to local health centers to identify and enroll eligible Muslim women. Simple random sampling was used to select 43 Muslim women from each group who had participated voluntarily and had never undergone a Pap smear test before the study.

The sample size was calculated by considering the assumptions of 90% statistical power, 5% significance level (two-sided alpha), proportions (P1 = 0.92, P2 = 0.65) from a previous study that investigated "The effects of a motivated teaching program program on perception and cervical cancer screening rate among rural Thai women."[21], and 20% expected loss to follow-up. The total sample size was 86 participants from the calculation, with 43 participants for each group. However, 3 participants dropped out during the follow-up period, and only 83 participants (intervention = 42, control = 41) completed the study.

### Intervention

The participants were divided into two groups: Muslim women from the Mueang Surat Thani district participated in the Edutainment Program of the intervention group, and Muslim women from the Chaiya district participated in the standard care of the control group. The Edutainment Program simultaneously provides information for education and entertainment. The program was based on the HBM theory to improve the knowledge and perception of cervical cancer screening and change the behavior of undergoing cervical cancer screening among Muslim women in the intervention group. The program was designed as a 4-week intervention with one session per week for 60 min. The Edutainment Program for Muslim audiences was developed using four components: video clips, folk songs, short films, and a reminder program. We developed a script for the short film and lyrics for the folk song based on the HBM, which included perceived susceptibility, severity, benefits, and barriers. Additionally, a reminder program for the intervention group using folk songs was broadcasted to the community every morning and evening and After the intervention 4 week, the research team conducted a follow-up program by providing information directly to the participants in their homes in their native language. Table 1 outlines the specifics of the Edutainment Program.

**Table 1:** Details of the Edutainment Program based on the Health Belief Model (HBM) [22]

Edutainment sessions	Materials	HBM constructs	Objectives	Teaching method	Session content
Session 1	Video Clip 1	Knowledge	To improve knowledge of cervical cancer	- Question and Answer - Discussion	General knowledge about cervical cancer including definition, types, stages, risk factors, signs and symptoms, and diagnosis
Session 2	Video Clip 2	Knowledge	To improve knowledge of the cervical cancer screening test	- Question and Answer - Discussion	Information about the procedure of the cervical cancer screening test
Session 3	Folk Song	- Perceived Susceptibility - Perceived Severity - Perceived Benefit - Perceived Barrier	To improve perception	- Listen to Folk Song - Sing the song - Discussion about lyrics - Group discussion - Brainstorming	Making susceptible to the risk of cervical cancer, identifying symptoms and warning signs as well as the severity and seriousness of the disease,and understanding the benefits of cervical cancer screening test
Session 4	Short Film	- Perceived Susceptibility - Perceived Severity - Perceived Benefit - Perceived Barrier	To improve perception	- Watch Short Film - Group discussion - Brainstorming - Using motivations	Talk about the susceptibility, severity, and benefits of undergoing a cervical cancer screening test, its effects on physical and mental health, the advantages of early detection through screening, and provide motivation to undergo screening in order to reduce perceived barriers

The participants in the control group did not receive any intervention apart from standard care, which included general knowledge of cervical cancer and information about cervical cancer screening from healthcare providers. The validity of the tool was assessed using a content validity test and content validity index (CVI). The intervention program was presented to three experts—a health education expert from College of public health sciences Chulalongkorn University, an epidemiology expert from College of public health sciences Chulalongkorn University and an Obstetrician-gynecologist from Surat Thani Hospital. They were asked to provide feedback based on the study objectives and the relevance of the content. Based on the feedback received from the experts, further modifications were made to the intervention program. This resulted in a content validity test score of 0.87 and a content validity index of 0.79. To ensure the reliability of the tool, and each construct had internal consistency reliability with Cronbach’s alpha of 0.71 it was administered to 30 Muslim women from the study population who matched the demographic variables of the sample group. Feedback was collected from all program participants through interviews prepared by the Reseacher team. The feedback from the program participants was carefully reviewed and used to enhance the content, increase engagement, and modify teaching methods to maximize effectiveness for the program participants.

### Measures

The participants completed a semi-structured questionnaire at baseline, post-intervention, and at 3-month follow-up through face-to-face interviews. The questionnaire, which was administered by the researcher, was written in Thai. The knowledge and perception questions were adapted from constructs used in similar studies. Shojaeizadeh et al. (2011) investigated "The Effect of an Educational Program on Increasing Cervical Cancer Screening Behavior among Women in Iran Applying the Health Belief Model," while Maneechot (2017) examined "The effects of a motivated teaching program on perception and cervical cancer screening rate among rural Thai women" [21, 23].

After holding research team meetings, and validity and reliability measurement, final questionnaire combination of was compiled and used. The knowledge questions had internal consistency reliability with a Cronbach’s alpha of 0.85 and consisted of 15 items (score range 0 – 15), with one score for each correct response. The perception questions based on the HBM constructs consisted of 15 items which included perceived susceptibility (4 items, score range 4—20), perceived severity (3 items, score range 3 – 15), perceived benefits (5 items, score range 5 – 25), and perceived barriers (3 items, score range 3—15) on five-point Likert scale (totally agree = 5, agree = 4, undecided = 3, disagree = 2, strongly disagree = 1), and each construct had internal consistency reliability with Cronbach’s alpha of 0.71, 0.71, 0.85, and 0.86, respectively. Uptake of the cervical cancer screening test was a binary outcome, in which the participants were categorized as having undergone cervical cancer screening at the time of assessment.

### Statistical analysis

Data were analyzed using the statistical package for social sciences (SPSS) (version 22.0; IBM Corp., Armonk, NY, USA) at a significance level of 0.05. Descriptive statistics such as frequency, percentage, mean, and standard deviation were used to summarize the data. The chi-square test of independence (or Fisher’s exact test) for categorical data and the independent sample t-test for continuous data were used to compare baseline characteristics between groups. Repeated-measures analysis of variance (ANOVA) was used to analyze the effect of the Edutainment Program on knowledge and perception based on the HBM constructs among Muslim women between and within groups at baseline, post-intervention, and at 3-month follow-up.

## Results

Table 2 summarizes the sociodemographic characteristics of participants by study group. Among the 83 Muslim women, more than one-third had a secondary school level of education (38.55%), followed by primary school level (26.51%). Most participants in this study (93.98%) were married. Over one-third of Muslim women were self-employed (37.35%) and another one-third were housewives (34.94%). Their average monthly income was around 5,000–8,000 baht, and most (84.34%) received universal health insurance coverage. The reported age at first delivery was between 21–25 years (34.94%), followed by ≤ 20 years (32.53%). Majority had two deliveries (38.96%), with two live children (42.86%). In addition, 7.23% of the women reported having no children because of abortion. Most participants received information about cervical cancer through television (20.48%); however, more than half (51.81%) did not receive any information. The baseline characteristics were similar between the two groups.

**Table 2:** Sociodemographic characteristics of participants by study groups (*N* = 83)

Variables	Intervention group (*N* = 42) n (%)	Control group (*N* = 41) n (%)	*p*-value
**Educational level**			0.154^b^
Uneducated	-	2 (4.88)	
Primary School	12 (28.57)	10 (24.39)	
Secondary School	18 (42.86)	14 (34.15)	
High school	10 (23.81)	7 (17.07)	
Higher than high school	2 (4.76)	8 (19.51)	
**Marital status**			0.172^b^
Married	41 (97.62)	37 (90.24)	
Widowed/Divorced	1 (2.38)	4 (9.76)	
**Occupation**			0.192ª
Housewife	18 (42.86)	11 (26.83)	
Merchant	12 (28.57)	11 (26.83)	
Self-employed	12 (28.57)	19 (46.34)	
**Income**			0.473ª
< 5,000 baht	9 (21.43)	12 (29.27)	
5,000—8,000 baht	13 (30.95)	14 (34.15)	
8,001 -11,000 baht	12 (28.57)	6 (14.63)	
≥ 11,001 baht	8 (19.05)	9 (21.95)	
**Health insurance**			0.799ª
Universal Coverage	35 (83.33)	35 (85.37)	
Social Security	7 (16.67)	6 (14.63)	
**Age at first delivery**			0.188^b^
≤ 20 years	16 (38.10)	11 (26.83)	
21–25 years	17 (40.48)	12 (29.27)	
26–30 years	5 (11.90)	8 (19.51)	
31–35 years	1 (2.38)	6 (14.63)	
> 35 years	3 (7.14)	4 (9.76)	
**Number of delivery**			0.071^b^
No	3 (7.10%)	3 (7%)	
One	1 (19.26)	13 (34.21)	
Two	17 (43.59)	13 (34.21)	
Three	13 (33.33)	7 (18.42)	
Four and above	4 (12.82)	5 (13.16)	
**Received information about cervical cancer**			0.206^b^
Never	26 (61.90)	17 (41.46)	
Newspaper	5 (11.90)	3 (7.32)	
TV	5 (11.90)	12 (29.27)	
Radio	2 (4.76)	1 (2.44)	
Internet	3 (7.14)	5 (12.20)	
Other	1 (2.38)	3 (7.32)	

^a^Chi-square test

^b^Fisher’s Exact test

Table 3 presents the results of the repeated-measures ANOVA to analyze the effect of the Edutainment Program on the knowledge of Muslim women between and within groups. There was a statistically significant difference between the intervention and control groups (*p* < 0.001). Within-subjects analysis revealed that the Edutainment Program resulted in significant changes in the mean knowledge of cervical cancer over the three time points (*p* < 0.001). The post-hoc pairwise comparison using Bonferroni correction indicated statistically significant differences between the intervention and control groups post-intervention and at 3-month follow-up (*p* < 0.001).

**Table 3:** Repeated measures ANOVA of knowledge about cervical cancer between and within groups (N = 83)

Knowledge	ss	df	MS	F-test	*P*-value
***Between subjects***					
Intervention	1428.393	1	1428.393	133.738	< 0.001*
Error (between-group error)	865.125	81	10.681		
***Within subjects***					
Time	801.422	1.559	514.054	253.798	< 0.001*
Intervention × time	826.948	1.559	530.427	261.882	< 0.001*
Error (within-group error)	255.775	126.281	2.025		

^*^Statistical significance at *p* < 0.05

Table 4 describes the results of the repeated measures ANOVA analyzing the effect of the Edutainment Program on perceptions based on the HBM constructs of Muslim women between and within groups. There was a statistically significant difference between the intervention and control groups (*p* < 0.001). The within-subjects analysis showed that the Edutainment Program resulted in significant changes in mean perception based on the HBM constructs regarding cervical cancer screening over the three time points (*p* < 0.001). The post-hoc pairwise comparison using Bonferroni correction indicated statistically significant differences between the intervention and control groups post-intervention and at 3-month follow-up (*p* < 0.001).

**Table 4:** Repeated measures ANOVA of perception based on the HBM constructs between and within groups (*N* = 83)

Perception based on HBM constructs	ss	df	MS	F-test	*P*-value
**Between subjects**					
Intervention	26.246	1	26.246	27.402	< 0.001*
Error (between-group error)	77.583	81	0.958		
**Within subjects**					
Time	28.706	1.158	24.780	130.821	< 0.001*
Intervention × time	17.457	1.158	15.069	79.554	< 0.001*
Error (within-group error)	17.774	93.836	0.189		

^*^Statistical significance at *p* < 0.05

Table 5 shows the percentage uptake of cervical cancer screening tests between the intervention and control groups at the three time points. At baseline, there was no difference in the percentage of cervical cancer screening tests performed between the intervention and control groups. The percentage of cervical cancer screening tests in the intervention group increased after implementation of the intervention program. The intervention group reported 90.47% of cervical cancer screening tests, which was nearly twice that of the control group (51.21%).

**Table 5:** Percent uptake of cervical cancer screening tests between the intervention and control groups (*N* = 83)

Time points	Intervention group (*N* = 42) n (%)	Control group (*N* = 41) n (%)
Baseline	5 (11.90)	6 (14.63)
Post-intervention	27 (64.28)	10 (24.39)
3-month follow-up	6 (14.28)	5 (12.19)
**Total**	**39 (90.47)**	**21 (51.21)**

## Discussion

The Edutainment Program aimed to improve the knowledge of cervical cancer and perceptions based on the HBM constructs about cervical cancer screening among Muslim women in Thailand. This study demonstrated that the Edutainment Program was successful in improving knowledge of cervical cancer. There were differences in knowledge scores before and after the intervention program. In our Edutainment Program, Muslim women may be encouraged to learn more about cervical cancer and the importance of cervical cancer screening tests. The Edutainment Program comprised watching video clips, listening to folk songs, and watching short films. The researcher developed each component of the program based on the culture and traditions of Muslims and South Asians. After the intervention sessions, the research team implemented a follow-up reminder program by personally delivering information to the participants in their homes using their native language. This is similar to house visits conducted by public health workers in other projects [24, 25]. This assists in connecting with the participants and motivates them to respond to any questions they may have regarding cervical cancer. These findings are consistent with those of a previous study that involved women in Malaysia and used educational talks, video displays, and experience-sharing sessions and reported that the results obtained by the actions of public health workers and multimedia were comparable [26]. Furthermore, an educational intervention that included lectures, discussions, videos, and leaflets was employed in a study on women in Ghana. In that study, health education was provided in churches, and it was found that health education interventions were critical in improving women’s knowledge of cervical cancer [27]. This is consistent with a previous study revealing that educational interventions improved the knowledge of women regarding cervical cancer in the experimental group [28, 29]. Additionally, a randomized community trial in Malaysia found that educational talks, video shows, and experience-sharing sessions significantly increased women’s knowledge in the intervention group [26].

The findings revealed that perceptions based on the HBM constructs changed over the three time periods in the intervention group. This demonstrates how duration over two months affects perception. Based on our program, the perception of the participants increased after talking with the researcher in the local language, which encouraged Muslim women to easily understand the information. They learned about cervical cancer by watching a video of the Edutainment Program, which simulated a discussion on cervical cancer between Muslim women and physicians. This tool aims to show that doctor-patient interaction remains uninterrupted. The main components of the folk songs in our Edutainment Program were launched when the participants performed routine activities, such as praying. Muslims developed the folk songs of the Edutainment Program using the melodies of Muslim folk songs. The short film of the Edutainment Program was designed based on the construct of perception in the HBM. Some studies found that perceived susceptibility is low among women in intervention groups [30]. A study of Saudi women reported that they perceived themselves as having low susceptibility to cervical cancer [31]. The timing of the intervention affected participants' perceptions of how cervical cancer would adversely affect their health. One of the key achievements in stimulating the participants’ perceptions was the researcher's on-site face-to-face action. A previous study found that receiving advice from healthcare providers was a common facilitator stimulating participants’ perceptions [32]. However, our findings differ from those of prior studies, which indicated no statistically significant differences in perceived susceptibility scores between the pre-and post-tests [27]. This difference can be explained by the implementation of the Edutainment Program and number of follow-up visits. This is similar to the findings of a systematic review of women in Southeast Asia, which found that embarrassment was one of the main barriers to cervical cancer screening test [32, 33]. Religious concerns were also a main barrier to the perception of women undergoing cervical cancer screening in this study [34–36]. This finding was supported by previous studies on breast and cervical cancer which reported that culture and values may have an impact on how HBM constructs affect breast and cervical cancer screening [37–39]. Although the study provided evidence for the effectiveness of the edutainment intervention, there were limitations such as the potential influence of other sources of information on both the intervention and control groups. Nonetheless, the HBM usage was a strength of this study, as it enabled the assessment of attitudes and behavior, and the Edutainment Program was beneficial in encouraging healthy behaviors among rural and underserved women.

### Limitation and recommendations

This study had three limitations. First, the study areas of the intervention and control groups were rural. Because of the differences in lifestyle, sociodemographic, and economic conditions between urban and rural areas, generalization of the findings is not possible. Second, it could be applied to Muslim women in Southern Thailand since the study employed an intervention using the local language. Finally, participants completed a 4-week Edutainment Program and a 3-month follow-up. This study program was only of short duration; future studies should adopt the program with a long follow-up period (six months) after it was completed to assess the sustainability of the program.

### Supplementary Information

Supplementary Material 1.

## Data Availability

Data for this study were extracted from a Ph.D. dissertation at the College of public health sciences Chulalongkorn University All data generated during and/or analyzed. during the study are available by the correspondent author on reasonable. request.

## Footnotes

1 HPV = Human Papilloma Virus.

## References

[1] Bruni L (2023). ICO/IARC Information Centre on HPV and Cancer (HPV Information Centre). Human Papillomavirus and Related Diseases in the World. Summary Report 10 March 2023..

[2] Small W (2017). Cervical cancer: a global health crisis. Cancer.

[3] Ferlay J (2020). Global cancer observatory: cancer today.

[4] Al-Hammadi FA (2017). Limited understanding of pap smear testing among women, a barrier to cervical cancer screening in the United Arab Emirates. Asian Pac J Cancer Prev.

[5] Lofters AK (2017). Cervical cancer screening among women from muslim-majority countries in Ontario. Canada Cancer Epidemiol Biomarkers Prev.

[6] Parkin DM (2005). Global cancer statistics, 2002. CA Cancer J Clin.

[7] Brink AA (2005). Clinical relevance of human papillomavirus testing in cytopathology. Cytopathology.

[8] Al Sairafi M, Mohamed FA (2009). Knowledge, attitudes, and practice related to cervical cancer screening among Kuwaiti women. Med Princ Pract.

[9] Laovutthanon P, Imsamran  V (2018). Guidelines for screening, diagnosis, and treatment of cervical cancer. N.C.I. Academic Support Unit, Editor..

[10] Office, S.T.P.P.H., Cervical cancer patient statistics for the year 2015–2019 2019, Surat Thani Provincial Public Health Office.

[11] Widiasih R, Nelson K (2018). Muslim husbands’ roles in women’s health and cancer: the perspectives of muslim women in Indonesia. Asian Pac J Cancer Prev.

[12] Padela AI (2014). Associations between religion-related factors and cervical cancer screening among Muslims in greater chicago. J Low Genit Tract Dis.

[13] Goldie SJ (2005). Cost-effectiveness of cervical-cancer screening in five developing countries. N Engl J Med.

[14] Peirson L (2013). Screening for cervical cancer: a systematic review and meta-analysis. Syst Rev.

[15] Siu AL (2016). Screening for Breast Cancer: U.S. Preventive services task force recommendation statement. Ann Intern Med.

[16] Pew-Research-Center, The Future of World Religions: Population Growth Projections, 2010-2050. 2015.

[17] Underwood SM, Shaikha L, Bakr D (1999). Veiled yet vulnerable. Breast cancer screening and the Muslim way of life. Cancer Pract.

[18] Padela AI, Curlin FA (2013). Religion and disparities: considering the influences of Islam on the health of American Muslims. J Relig Health.

[19] Padela AI (2012). Religious values and healthcare accommodations: voices from the American Muslim community. J Gen Intern Med.

[20] Matin M, LeBaron S (2004). Attitudes toward cervical cancer screening among Muslim women: a pilot study. Women Health.

[21] Maneechot P, Songwatthana P, Kritcharoen S (2013). The effects of motivated teaching program on disease perception and cervical cancer screening rate among Rural Thai women. Srinagarind Medical Journal.

[22] Glanz K, Rimer BK, Viswanath K. Health behavior and health education: theory, research, and practice. San Francisco, CA: Wiley; 2008. www.josseybass.com.

[23] Shojaeizadeh D (2011). The effect of educational program on increasing cervical cancer screening behavior among women in Hamadan, Iran: applying health belief model. J Res Health Sci.

[24] Choi Y (2022). Uptake and correlates of cervical cancer screening among women attending a community-based multi-disease health campaign in Kenya. BMC Womens Health.

[25] Altunkurek ŞZ (2022). Knowledge and attitudes of healthcare professionals working in a training and research hospital on early diagnosis of cervical cancer (a Somalia example): cross-sectional study. BMC Womens Health.

[26] Romli R (2020). Effectiveness of a health education program to improve knowledge and attitude towards cervical cancer and pap smear: a controlled community trial in Malaysia. Asian Pac J Cancer Prev.

[27] Ebu NI (2019). Impact of health education intervention on knowledge and perception of cervical cancer and screening for women in Ghana. BMC Public Health.

[28] Eghbal SB (2020). Evaluating the effect of an educational program on increasing cervical cancer screening behavior among rural women in Guilan. Iran BMC Women's Health.

[29] Bevilacqua KG (2022). Cervical cancer knowledge and barriers and facilitators to screening among women in two rural communities in Guatemala: a qualitative study. BMC Womens Health.

[30] Bebis H (2012). Effect of health education about cervical cancer and papanicolaou testing on the behavior, knowledge, and beliefs of Turkish women. Int J Gynecol Cancer.

[31] Aldohaian AI, Alshammari SA, Arafah DM (2019). Using the health belief model to assess beliefs and behaviors regarding cervical cancer screening among Saudi women: a cross-sectional observational study. BMC Womens Health.

[32] Chua B (2021). Barriers to and facilitators of cervical cancer screening among women in Southeast Asia: a systematic review. Int J Environ Res Public Health.

[33] Gottschlich A (2019). Barriers to cervical cancer screening and acceptability of HPV self-testing: a cross-sectional comparison between ethnic groups in Southern Thailand. BMJ Open.

[34] Hausiku L, Kouame K, Aboua YG (2022). Perceptions and attitude of women of Luderitz, Namibia on Pap smear and cervical cancer prevention. BMC Womens Health.

[35] Negash BA, Bayu NH, Woretaw AW (2023). Knowledge, attitude, and associated factor towards cervical cancer prevention among primary and secondary school female teachers in Gondar town, North West Ethiopia, 2022. BMC Womens Health.

[36] Zhang B (2023). Knowledge, willingness, uptake and barriers of cervical cancer screening services among Chinese adult females: a national cross-sectional survey based on a large e-commerce platform. BMC Womens Health.

[37] Chongthawonsatid S (2017). Inequity of healthcare utilization on mammography examination and Pap smear screening in Thailand: Analysis of a population-based household survey. PLoS ONE.

[38] Hajian-Tilaki K, Auladi S (2014). Health belief model and practice of breast self-examination and breast cancer screening in Iranian women. Breast Cancer.

[39] Enyan NIE (2022). Correlates of cervical cancer screening participation, intention and self-efficacy among Muslim women in southern Ghana. BMC Womens Health.

