# Supplementary material for: The edutainment program on knowledge, perception, and uptake of cervical cancer screening among Muslim women in Southern Thailand: a quasi experimental study
Source: BMC Public Health. 2024 Jul 6;24:1803. doi: 10.1186/s12889-024-19287-y (PMC11227195; doi:10.1186/s12889-024-19287-y)
Supplement: Supplementary file 1 — Supplementary Material 1. [file 12889_2024_19287_MOESM1_ESM.pdf]

Appendix Questionnaire (English version)

Part 1 Socio-demographic Characteristics

Explanation: Fill in the blank or mark ✓ in the blank that consist with your data.

1.1 Marital status

( ) Married ( ) Widow/Divorce

1.3 Health insurance

( ) Universal Coverage Scheme

( ) Social Security Scheme

( ) Government Or State Enterprise Officer

( ) Other………………

1.4 Education level

( ) No education

( ) Primary school

( ) Secondary school

( ) High school

( ) High vocational certificate / Diploma

( ) Bachelor's degree

( ) Master degree

1.5 Occupation ( ) Housewife ( ) Merchant ( ) fisherman ( ) self-employed

( ) Other

1.6 How many children do you have? ( ) 0 ( ) 1 ( ) 2 ( ) 3 ( ) more than 3

1.7 How many time to Pregnancy? ( ) 0 ( ) 1 ( ) 2 ( ) 3 ( ) more than 3

1.8 Do you have some relatives with cervical cancer?

( ) Yes Who? ____________________

( ) No

1.8 In the last 1 months, can you remember seeing or hearing anything in the media, information in community about the signs and symptoms of cervical cancer? If yes, where did you hear or see it? (Please select all that apply)

( ) Newspaper ( ) TV ( ) Radio ( ) Website ( ) Other

( ) Never

Part 2 Cervical cancer Knowledge.

Please mark ✓ in the blank that match your opinion as much as possible.

Item	Questions	True	False
2.1	Itchiness in the vaginal area can be a sign of cervical cancer		
2.2	Cervical cancer is most common for women in their 20s		
2.3	Cervical cancer is the main cause of cancer related death among women		
2.4	Cervical cancer is often found at an early stage due to obvious symptoms		
2.5	You can get cervical cancer from deep kissing		
2.6	You can get cervical cancer if u have multiple sexual partners.		
2.7	Married Women can get cervical cancer more than single women.		
2.8	You can get cervical cancer from unprotected sexual intercourse		
2.9	If you have many children, you can get cervical cancer.		
2.10	Target population of cervical cancer screening test is women age above 60 years old		
2.11	You can uptake cervical cancer screen test at the hospital only.		
2.12	You should uptake of cervical cancer screening test If you have abnormal vaginal discharge.		
2.13	You can uptake cervical cancer screening test while you are having menstruation.		
2.14	Even if you don't have symptoms But you are over 30 years old, you can uptake cervical cancer screening test.		
2.15	Cervical cancer cannot treatment.		

Part 3 Health belief model Questionnaires.

Explanation: Mark ✓ in the blank are common recommendations.

To what extent do you agree or disagree with the following statements?	Strongly Disagree (1)	Disagree (2)	Undecided (3)	Agree (4)	Strongly agree. (5)
**Perception of Susceptibility**					
3.1 Women with multiple sexual partners are at risk of cervical cancer more than women with single sexual partners.					
3.2 Women who get married at a young age have a risk of Cervical cancer more than women married at older.					
3.3 Women who have children are get a risk of cervical cancer.					
3.4 Women age above 30 years risk of cervical cancer					
3.5 Cervical cancer sporadically can spread to other organs					
3.6 Cervical cancer would make a women's life very difficult.					
3.7 Cervical cancer may lead to having a hysterectomy.					
**Perception of Benefits**					
3.8Getting a Pap test at least 1 time of year can early detection of cervical cancer.					
3.9 A Pap test cervical cancer in the stage Initially,there are more opportunities for treatment.					
3.10 uptake of cervical cancer should go Annual checkup since no symptoms, better than check When the symptoms are abnormal					

To what extent do you agree or disagree with the following statements?	Strongly Disagree (1)	Disagree (2)	Undecided (3)	Agree (4)	Strongly agree. (5)
3.11 Up take of cervical cancer screening can reduce the risk and will reduce the cost of treatment.					
3. 12 seeing a doctor every time when you have vaginal discharge or irregular menstruation bleeding can reduce cause cervical cancer.					
**Perception of Barriers**					
3.13 It is difficult to get a Pap test because Pap smear conflict with religion.					
3.14 Getting a Pap test is embarrassment.					
3.15 I do not have time to get a Pap test.					
